# Cortical and Cerebellar Oscillatory Responses to Postural Instability in Parkinson's Disease

**DOI:** 10.3389/fneur.2021.752271

**Published:** 2021-11-04

**Authors:** Taylor J. Bosch, Stefan Kammermeier, Christopher Groth, Matt Leedom, Elizabeth K. Hanson, Patti Berg-Poppe, Arun Singh

**Affiliations:** ^1^Division of Basic Biomedical Sciences, Sanford School of Medicine, University of South Dakota, Vermillion, SD, United States; ^2^Center for Brain and Behavior Research, University of South Dakota, Vermillion, SD, United States; ^3^Department of Neurology, Ludwig Maximilian University, Munich, Germany; ^4^Department of Neurology, University of Iowa, Iowa City, IA, United States; ^5^Avera Therapy, Sioux Falls, SD, United States; ^6^Department of Communication Sciences and Disorders, University of South Dakota, Vermillion, SD, United States; ^7^Department of Physical Therapy, University of South Dakota, Vermillion, SD, United States

**Keywords:** postural control, balance, EEG, oscillations, theta, cerebellum

## Abstract

**Introduction:** Posture and balance dysfunctions critically impair activities of daily living of patients with progressing Parkinson's disease (PD). However, the neural mechanisms underlying postural instability in PD are poorly understood, and specific therapies are lacking. Previous electrophysiological studies have shown distinct cortical oscillations with a significant contribution of the cerebellum during postural control tasks in healthy individuals.

**Methods:** We investigated cortical and mid-cerebellar oscillatory activity *via* electroencephalography (EEG) during a postural control task in 10 PD patients with postural instability (PDPI+), 11 PD patients without postural instability (PDPI–), and 15 age-matched healthy control participants. Relative spectral power was analyzed in the theta (4–7 Hz) and beta (13–30 Hz) frequency bands.

**Results:** Time-dependent postural measurements computed by accelerometer signals showed poor performance in PDPI+ participants. EEG results revealed that theta power was profoundly lower in mid-frontal and mid-cerebellar regions during the postural control task in PDPI+, compared to PDPI– and control participants. In addition, theta power was correlated with postural control performance in PD subjects. No significant changes in beta power were observed. Additionally, oscillatory changes during the postural control task differed from the resting state.

**Conclusion:** This study underlines the involvement of mid-frontal and mid-cerebellar regions in postural stability during a balance task and emphasizes the important role of theta oscillations therein for postural control in PD.

## Introduction

Motor symptoms of Parkinson's disease (PD) include full body motor dysfunctions in balance, posture, and gait. These are particularly prevalent in advanced stages as well as in older PD patients (1–5). Postural instability is a common and disabling full body motor feature of PD, which is likely to affect more than 80% of PD patients in the disease progress (6). Approximately 60% of PD patients experience at least one disease-related fall and 40% have frequent falls related to postural instability (7).

In the clinical setting, PD is diagnosed based on the cardinal motor features of bradykinesia and rigidity, with non-obligatory contributing motor symptoms like postural instability or tremor. Non-motor symptoms, particularly cognitive deficits worsening with disease progression, add to overall disability and increase the risk of postural control disturbances (8–10). In healthy people, the postural control system integrates visual, proprioceptive, and vestibular sensory information; studies indicate that this integration of relevant sensory information may be critically disrupted in PD (11, 12).

Regarding current therapeutic interventions, levodopa and deep-brain stimulation (DBS) therapies do not effectively improve balance control in PD in the disease progress and no long-term efficacy of either therapy has been proven for PD postural instability (13–16). This instability may be related to dopaminergic disruption to the neural integration of jerk or sway. Dopamine regulation systems may be changed in the long term by levodopa, as demonstrated for PD dyskinesias (17). Histoanatomical degeneration of basal forebrain cholinergic neurons has been noticed in PD brains (18), a system associated with executive functions and another likely contributor to PD postural impairments (19). Additionally, the pedunculopontine nucleus provides cholinergic inputs to the thalamus, cerebellum, and the basal ganglia (20), and degeneration of large cholinergic neurons has been shown in advanced PD patients with motor abnormalities (21–23).

Regarding neural network activity patterns, our previous reports have demonstrated the presence of smaller theta-band (4–7 Hz) oscillations in the frontal regions of PD patients during cognitive processing and lower-limb motor tasks (24–26). However, the underlying basal forebrain network dysfunction and neural mechanism, by which postural instability could emerge in PD, are not well-known.

Most studies of PD postural impairments have focused on frontal and basal ganglia regions, whereas the activity in the cerebellar area has often been overlooked. Previous evidence suggests a combined role of frontal and cerebellar regions for balance dysfunction in PD (23, 27–29). Functional and morphological modulation in the cerebellar region can be associated with motor and non-motor symptoms (30–32). A compensatory effect of the cerebellum may help motor and non-motor functions in PD: at least in the early stage of the disease, activity in the cortico-cerebellar-thalamic-cortical circuit correlates with the severity of symptoms, suggesting a strong compensatory effect (33). Further into the advanced stage of the disease, this effect may diminish and might even contribute to the balance dysfunction.

Our knowledge about the role of cerebellar oscillations in PD remains limited, due to technical obstacles when recording cerebellar oscillations. Previous reports have shown the feasibility of cerebellar oscillation recordings in humans (34–36). Therefore, in addition to cortical activity, it is crucial to investigate the role of cerebellar activity for postural instability as a potential neuromodulatory target to improve balance or other full body motor symptoms. The general feasibility of cerebellar activity modulation for specific brainstem dysfunctions has been proven, for e.g., upbeat and downbeat nystagmus disorders with 4-Aminopyridine (37, 38).

Here, we investigated cortical and mid-cerebellar oscillations in PD patients with and without postural instability during a postural challenge task to examine the combined role of frontal and cerebellar oscillations in postural control function. Given previous findings regarding low frequency frontal oscillations and lower-limb motor problems in PD, as well as known connectivity between frontal and cerebellar regions, we predicted that PD patients with postural instability would experience lower theta-band power over mid-frontal and mid-cerebellar regions compared to PD patients without postural instability and age-matched healthy controls.

## Materials and Methods

### Participants

A total of 36 participants (*n* = 21 PD patients; *n* = 15 healthy control subjects) were recruited for the current study. Patients were categorized into two groups: PD with postural instability (PDPI+; *n* = 10) and without postural instability (PDPI−; *n* = 11). All recruitment for the patients was based on the diagnostic criteria recommended by the United Kingdom PD Society Brain Bank. Participants provided written informed consent in accordance with the Declaration of Helsinki. All experimental protocols were approved by the University of South Dakota and the University of Iowa Institutional Review Boards. Severity of PD was assessed by the motor part of the Unified Parkinson's Disease Rating Scale (mUPDRS) (39). PDPI+ participants were selected on the basis of the following criteria: (a) their clinical balance score was greater or equal to five (CBS; sum of mUPDRS items (max. 16) # Leg Agility, # Arising from Chair, # Posture, and # Postural Stability); (b) PDPI+ status was clinically verified by a movement disorders specialist; (c) for subjective confirmation of PDPI+, an unassisted balance task (standing on the foam pad) was performed immediately prior to the study trials. All participants performed the NIH Toolbox Dimensional Change Card Sort (DCCS) test to demonstrate cognitive function (40).

Since we intended to examine every-day postural function and due to the potential fall hazard among unmedicated PD patients (7, 8, 12, 13), all PD participants were treated with their usual prescribed levodopa medication and performed the postural task during “ON” medication, without showing dyskinetic features. Clinical demographics were matched across groups and are summarized in Table 1.

**Table 1:** Demographic and clinical assessments.

**Measure**	**Control** **(***n*** = 15)**	**PD** **(***n*** = 21)**	**PDPI−** **(***n*** = 11)**	**PDPI+** **(***n*** = 10)**	**Control vs. PD**	**PDPI– vs. PDPI+**
					**Independent** ***t*****-test**	**Independent** ***t*****-test**
Gender (M/F)^$^	10/5	16/5	9/2	7/3	0.53	0.53
Age (years)	70.9 ± 8.7	68.0 ± 9.3	65.5 ± 10.8	70.7 ± 7	0.97 (0.34)	−1.31 (0.21)
DD (years)	–	4.6 ± 3.1	4.2 ± 3.0	5.0 ± 3.2	–	−0.60 (0.55)
LEDD (mg)	–	805 ± 458	725 ± 467	894 ± 456	–	−0.84 (0.41)
mUPDRS	–	15.1 ± 8.2	9.2 ± 6.3	21.5 ± 4	–	−5.28 (<0.001)^**^
CBS	–	6.3 ± 5.7	1.4 ± 1.4	6.8 ± 2	–	−7.37 (<0.001)^**^
DCCS	58.4 ± 14.6	48.8 ± 10.7	54.1 ± 11.8	42.9 ± 5.3	2.29 (0.029)^*^	2.75 (0.01)^**^

*DD, Disease duration; LEDD, Levodopa equivalent daily dose; CBS, Clinical Balance Score; mUPDRS, motor Unified Parkinson's Disease Rating Scale; DCCS, Dimensional Change Card Sort*.

$ *Chi-squared test. Values presented as mean ± standard deviation*.

*Statistics presented as t-value (p-value)*.

* *p < 0.05*;

** *p < 0.01*.

### Behavioral Data and Analysis

Participants stood quietly on the balance pad (15.5″ L × 12.5″ W × 2.5″ H size) with their feet placed equidistantly to the right and left of the center line of the pad (stance width = ~7.75″) and looked straight ahead during the postural control task. This balance pad was made of thermoplastic elastomer foam. They performed the task without any tactile support, yet a study aide posed behind the subjects would step in to prevent an imminent fall. A triaxial accelerometer (Brain Products) was attached to the left thigh, to collect mediolateral (ML; *Y*-axis) and anteroposterior (AP; *Z*-axis) signals (41, 42). Accelerometer sensors can be used for the assessment of balance and postural control in disease conditions (43, 44) and were plugged into the AUX port of the EEG amplifier (Brain Products) for simultaneous recording with the EEG signals. We processed acceleration signals in Matlab (MathWorks) to compute time-dependent changes to measure a more subtle reflection of postural control (44). We converted the unit of acceleration signals from “g” to “m/s^2^″ by multiplication with 9.8. We down-sampled data to 50 Hz and removed the gravitational factor *via* the Matlab “detrend” function. *Post-hoc* filtering employed a 4th order Butterworth filter with a band-pass 0.1–3 Hz. Subsequently, we computed the Euclidean mean signal from both *X* and *Y* axes, mean acceleration, root mean square values *via* the Matlab “rms” function and power of the acceleration power spectrum (0.1–3 Hz) by the “FFT” function. The analysis used both mediolateral and anteroposterior plane channels to compute an ellipse representing the mean explored limits of stability and its respective area under the curve (AUC) (45).

### EEG Data and Analysis

A 64-channel customized EEG cap (Easycap) in conjunction with the Brain Vision amplifier (Brain Products) was used to collect signals at hardware-filtered 0.1 Hz high-pass and a 500 Hz sampling rate from cortical and cerebellar regions in PDPI+, PDPI–, and age-matched control healthy subjects during independent undisturbed stance on the balance pad. The “Pz” electrode was used as reference; “Fpz” electrode was used as ground. Signals from the Fp1, Fp2, FT9, FT10, TP9, and TP10 channels were removed before the preprocessing steps due to regular contamination with mimical and masticatory artifacts, resulting in 59 channels. In addition, a custom mid-cerebellar electrode (Cbz) was placed over the posterior fossa that corresponds to medial aspects of lobules VII, VIII, and IX. EEG signals were divided into consecutive 3 s epochs. The signal from the reference electrode was retrieved using the average reference method. Bad channels and bad epochs were classified using the Matlab “FASTER” and “pop_rejchan” algorithms at default parameters (46). Residual traces of eye movements and other artifacts were removed using independent component analysis *via* the “ADJUST” algorithm which uses artifact-specific spatial and temporal characteristics to reject eye, muscle, and generic discontinuities. Spectral analysis was implemented on the epoched and preprocessed data using the “pwelch” function. We selected a 1-s time window as segment length, and the number of overlapping samples was set to 50% of the window length. We computed relative spectral power using the mean value from 0.1 to 50 Hz to avoid inter-subject variability. Spectral properties of the lateral motor cortical (C3 and C4), mid-frontal leg premotor area (Cz), and mid-cerebellar (Cbz) signals in the theta-band (4–7 Hz) and beta-band (13–30 Hz) were derived and compared between groups.

Signals from the cerebellar EEG electrodes were obtained from among the occipital regions of the 64-channel setup in accordance with previous studies (34–36). We compared the mid-cerebellar (Cbz) and mid-occipital (Oz) signals among all participants. Additional analyses compared the Cbz signals from the nearby muscle activity *via* EMG recording during resting-state in 5 PD patients. EMG electrodes were placed above the semispinalis capitis muscle. To differentiate between the postural control and the resting state EEG signals, we recorded EEG signals during a resting-state while participants were sitting on the chair with their eyes open. Resting EEG signals were processed similar to the postural control EEG signals. For both resting-state and postural control EEG data, we collected 3–4 min of continuous data which did not significantly differ in time across groups before and after preprocessing. Given the 3-s epoch length, this resulted in 60–80 epochs/trials per participant.

### Statistical Analysis

All statistical analyses were performed using the Statistical toolbox of Matlab. Initially, we performed independent *t*-tests to compare clinical demographics between PD patients and control subjects, PDPI+ and PDPI–. We performed one-way analysis of variance (ANOVA) tests to compare cognitive (DCCS scores), behavioral (acceleration, rms values, ellipse area under the curve, and power spectral values) and EEG (theta and beta power values) outcomes between all three groups and applied multiple comparisons tests using the Tukey–Kramer approach with an alpha level of <0.05. We measured the effect size with Eta^2^ (η^2^). We applied the Spearman correlation method to compute the relationship between two variables. Resting-state data were analyzed similar to the postural data. In order to compare between the postural task and resting-state activities, we used a 2 × 2 repeated measures ANOVA with a between-subjects variable (groups) and a within-subjects variable (rest vs. postural task).

We demonstrated the signal quality of the mid-cerebellar (Cbz) signal and volume conduction from the mid-occipital signal by computing the signal similarities between Cbz and Oz using the cross-correlation method to export the amplitude and phase values. A reference EMG lead among PD patients (*n* = 5) on the splenius capitis et cervicis nearby the Cbz lead was compared to the Cbz resting-state activity *via* correlation and cross-correlation methods. Moreover, to compare task-related activities between Cz, Oz, and Cbz, we first implemented a one-way ANOVA with multiple comparison tests for each group and subsequently performed 2 × 2 repeated measures ANOVA tests with a between-subjects variable (groups) and a within-subjects variable (electrodes) for theta power.

## Results

### Clinical and Behavioral Outcomes

We initially assessed differences in clinical scores between PDPI+, PDPI–, and healthy control participants (see Table 1 for details). The results from the one-way ANOVA assessing differences in cognitive function through the DCCS task revealed a main effect of “group” [*F*_(2,33)_ = 5.23, *p* = 0.011, η^2^ = 0.24; Supplementary Figure 1A]. Pairwise comparisons demonstrated a difference between PDPI+ and controls (*p* = 0.01) as well as a trend between PDPI+ and PDPI– participants (*p* = 0.09). No difference was observed between PDPI– and controls (*p* = 0.63). Further correlation analyses showed significant negative correlations between DCCS scores and clinical balance scores (rho = −0.8, *p* < 0.001; Supplementary Figure 1B), as well as disease severity assessed using the mUPDRS (rho = −0.78, *p* < 0.001; Supplementary Figure 1C). These findings underline the considerable interrelationship between cognitive function, postural instability, and disease severity.

**Figure 1:**
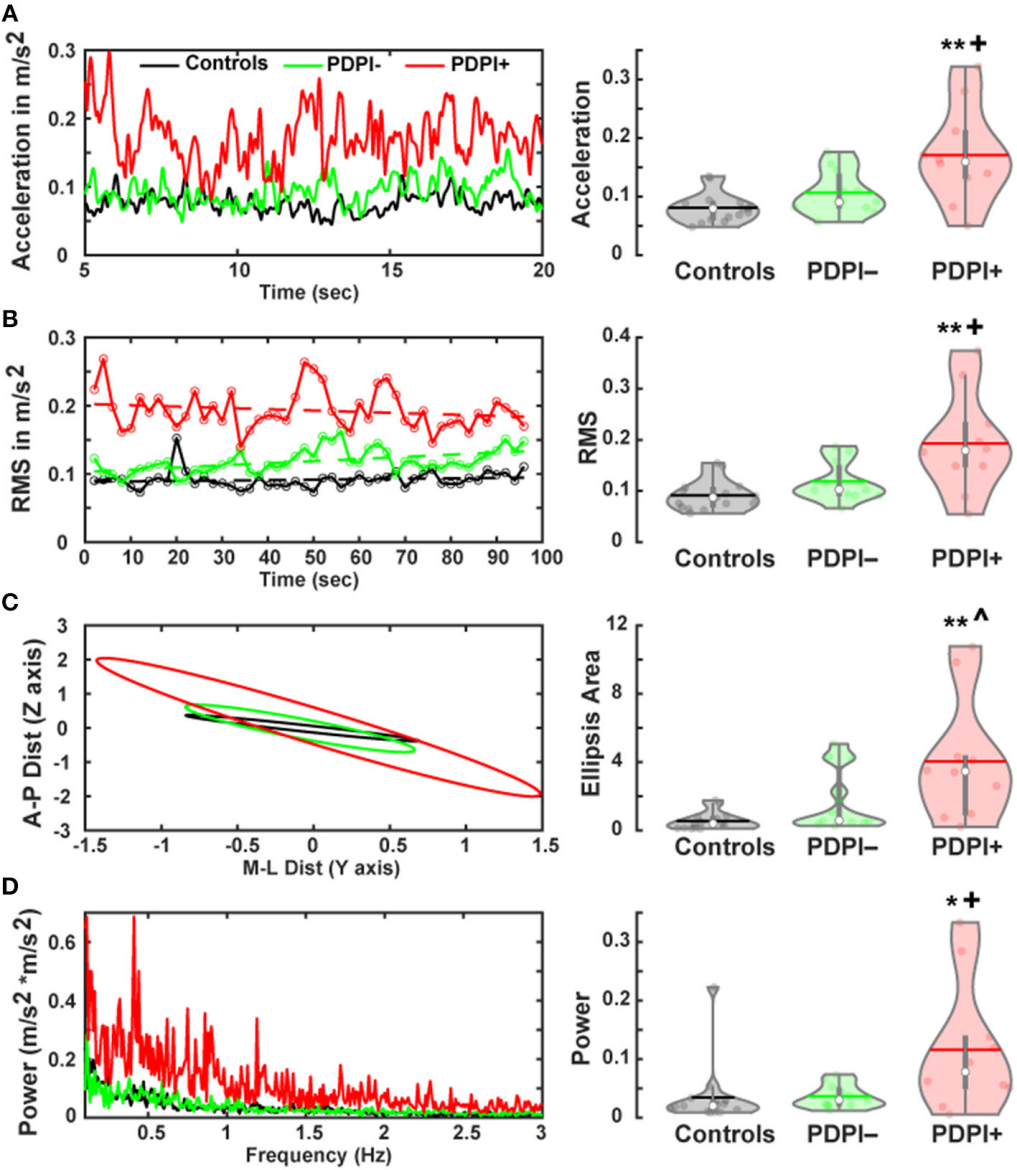
Parkinson's disease patients with postural instability (PDPI+) show multiple balance deficits during a postural control task. **(A)** Accelerometer signals of PDPI+ demonstrate increased mean acceleration computed through Euclidean mean signals (anteroposterior and mediolateral signals) and **(B)** increased magnitude of acceleration computed through the root mean square (RMS) of acceleration. **(C)** PDPI+ patients display increased limits of stability as indicated by area of the ellipsis computed using mean anteroposterior and mediolateral movement distances. **(D)** PDPI+ patients have increased mean spectral power of acceleration across the 0.1–3 Hz frequency band. ^**^p < 0.01 vs. controls, ^*^p < 0.05 vs. controls, ^+^*p* < 0.05 vs. PDPI–, ^∧^*p* < 0.06 vs. PDPI–. The horizontal lines and white circles in the violin plots represent the mean and median values, respectively.

A primary purpose of this study was to assess differences in postural stability between the three groups. Therefore, we performed multiple one-way ANOVAs on measures that assess different aspects of posture including the Euclidean mean signal to measure postural outcomes by mean acceleration. These results demonstrated a main effect of “group” [*F*_(2,33)_ = 9.66, *p* < 0.001, η^2^ = 0.37; Figure 1A]. Pairwise comparisons revealed differences between PDPI+ and controls (*p* < 0.01) and between PDPI+ and PDPI− (*p* = 0.02), but no difference between PDPI– and controls (*p* = 0.42). Subsequently, we used the root mean square to measure the magnitude of the acceleration traces. Similarly, we observed a main effect of “group” [*F*_(2,33)_ = 9.17, *p* = 0.001, η^2^ = 0.36; Figure 1B] with pairwise comparisons showing differences between PDPI+ and controls (*p* < 0.01) and between PDPI+ and PDPI− (*p* = 0.02), but not between PDPI– and controls (*p* = 0.49).

From the mediolateral and anteroposterior accelerometer signals we derived a two-dimensional ellipsis, representing the mean limits of stability. The area under the curve (AUC) for these plots demonstrated a main effect of “group” [*F*_(2,33)_ = 7.82, *p* = 0.002, η^2^ = 0.32; Figure 1C], as well as significant differences between PDPI+ and controls (*p* < 0.01) and between PDPI+ and PDPI− (*p* = 0.06), but not between PDPI− and controls (*p* = 0.34). The power of the acceleration power spectrum was computed between 0.1 and 3 Hz and compared across groups. Similar to aforementioned accelerometer results, a main effect of “group” was observed [*F*_(2,33)_ = 5.08, *p* = 0.012, η^2^ = 0.24; Figure 1D] with differences between PDPI+ and controls (*p* = 0.02) and between PDPI+ and PDPI– (*p* = 0.03), but not between PDPI− and controls (*p* = 1).

We then investigated possible associations between clinical balance scores and accelerometer data (Supplementary Figure 2). There was an association between clinical balance scores and mean acceleration (rho = 0.43, *p* = 0.05) and power of the acceleration power spectrum (rho = 0.46, *p* = 0.038). An association trend was observed for clinical balance scores with root mean square measures (rho = 0.41, *p* = 0.063). Overall, behavioral results underlined the expected differences between PDPI+ and PDPI– participants regarding postural instability.

### Mid-frontal and Mid-cerebellar Theta and Beta Oscillations

**Figure 2:**
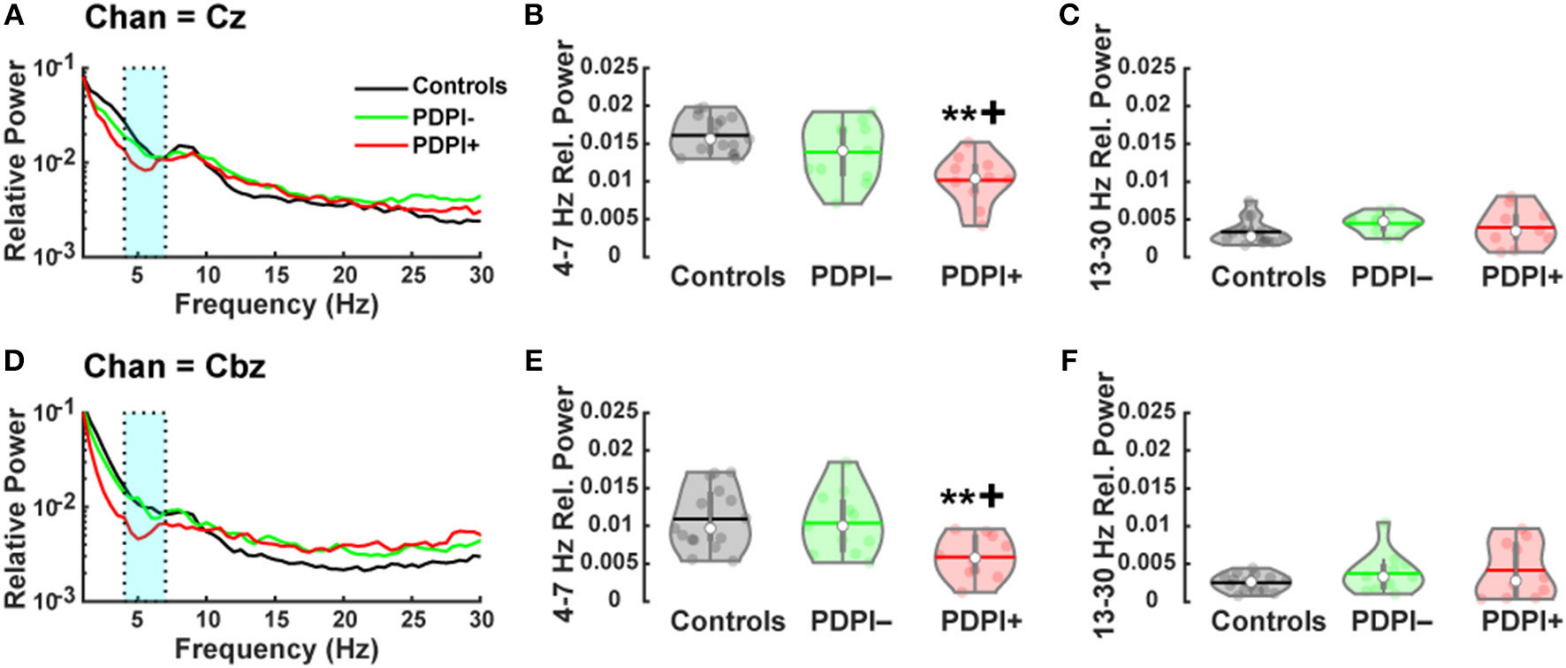
Parkinson's disease patients with postural instability (PDPI+) demonstrate lower mid-frontal and mid-cerebellar theta-band power during a postural control task. **(A)** Spectral power distribution for the mid-frontal Cz electrode. **(B)** PDPI+ exhibit decreased mid-frontal theta-band (4–7 Hz) power compared to PDPI– and healthy controls. **(C)** No difference is observed in the beta-band (13–30 Hz) over the mid-frontal Cz electrode. **(D)** Spectral power distribution for the mid-cerebellar Cbz electrode. **(E)** PDPI+ exhibit decreased mid-cerebellar theta-band power compared to PDPI– and healthy controls. **(F)** No difference is observed in the beta-band over the mid-cerebellar Cbz electrode. **(A–D)** Cyan box indicates the theta-band. ^**^p ≤ 0.01 vs. controls, ^+^*p* < 0.05 vs. PDPI–. The horizontal lines and white circles in the violin plots represent the mean and median values, respectively.

Given our previous studies showing lower mid-frontal and mid-cerebellar theta-band during cognitive and motor tasks (24, 26), we first examined differences between groups among the electrodes Cz and Cbz. Given the established relationship between beta-band activity and movement activity (25, 47), we also examined differences across the three groups for Cz and Cbz in the beta-band. The results from the one-way ANOVA examining the theta-band over Cz (Figure 2A) demonstrated a main effect of “group” [*F*_(2,33)_ = 10.25, *p* < 0.001, η^2^ = 0.38; Figure 2B]. Pairwise comparisons revealed significant differences between PDPI− and PDPI+ participants (*p* = 0.03) and between PDPI+ and controls (*p* < 0.01), but again no difference between PDPI− and controls (*p* = 0.21). For the beta-band, there were no effects of “group” at Cz [*F*_(2,33)_ = 1.21, *p* = 0.310, η^2^ = 0.07; Figure 2C].

Similarly, when examining the theta-band over mid-cerebellar Cbz (Figure 2D), the one-way ANOVA revealed a main effect of “group” [*F*_(2,33)_ = 5.86, *p* = 0.007, η^2^ = 0.26; Figure 2E] with pairwise comparisons showing differences between PDPI+ and controls (*p* = 0.01) and between PDPI+ and PDPI− participants (*p* = 0.03), but no difference between PDPI− and controls (*p* = 0.93). In the beta-band, no effect of any group was seen at the electrode Cbz [*F*_(2,33)_ = 1.39, *p* = 0.264, η^2^ = 0.08; Figure 2F]. Overall, our EEG results demonstrated that PDPI+ participants had lower mid-frontal and mid-cerebellar theta-band power when performing the postural control task. Furthermore, these changes were absent in the generally movement-related beta-band.

In addition to these bands, we also performed a one-way ANOVA to examine alpha-band differences across groups at the mid-frontal Cz and mid-cerebellar Cbz electrodes. At the mid-frontal Cz electrode, no main effect of group was observed [*F*_(2,33)_ = 0.09, *p* = 0.91]. Similarly, no main effect of group was observed at the mid-cerebellar Cbz electrode [*F*_(2,33)_ = 0.19, *p* = 0.83].

**Figure 3:**
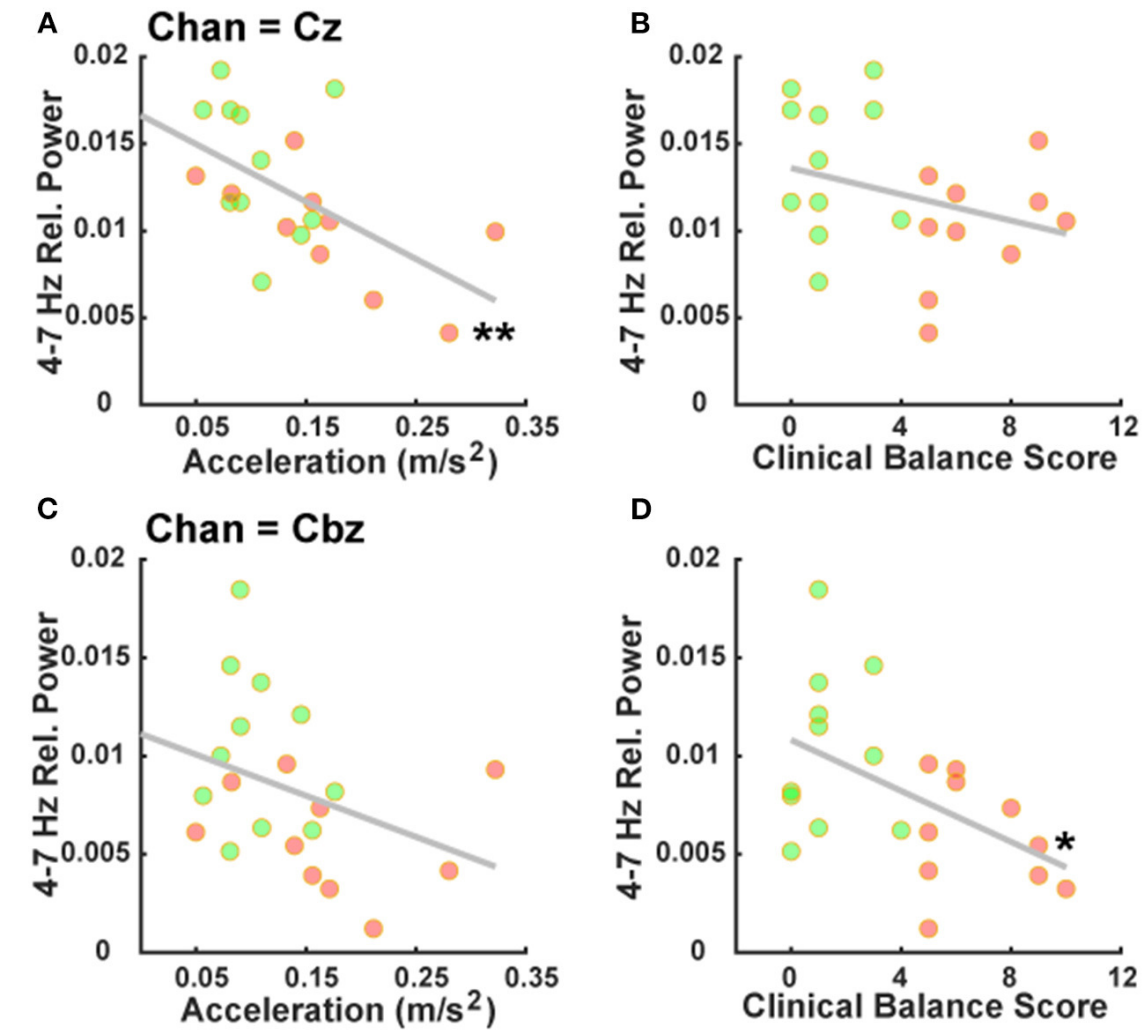
Mid-frontal and mid-cerebellar theta-band power values are associated with distinct measures of postural instability. **(A)** Decreased mid-frontal theta-band (4–7 Hz) power is significantly associated with higher mean acceleration during a postural control task. **(B)** Mid-frontal theta-band power is not significantly associated with clinical balance scores. **(C)** Mid-cerebellar theta-band power is not significantly associated with mean acceleration during a postural control task. **(D)** Decreased mid-cerebellar theta-band power is significantly associated with higher (worse) clinical balance scores. ^*^p < 0.05, ^**^p < 0.01. Green and red markers represent PDPI– and PDPI+ subjects, respectively.

Correlation analyses comparing theta-band power at our mid-frontal and mid-cerebellar electrodes with clinical balance scores and mean acceleration from the postural control task provided specific outcomes. Notably, theta-band power over the mid-frontal electrode Cz correlated with mean acceleration (rho = −0.61, *p* = 0.004; Figure 3A), but not with clinical balance scores (rho = −0.33, *p* = 0.15; Figure 3B). By contrast, theta-band power over the mid-cerebellar electrode Cbz did not correlate with mean acceleration (rho = −0.38, *p* = 0.09; Figure 3C), but did correlate with clinical balance scores (rho = −0.45, *p* = 0.04; Figure 3D). These results serve to reinforce our established group differences and suggest that mid-frontal and mid-cerebellar theta-band activity is related to multiple assessments of balance.

### Comparison Between Mid-frontal, Mid-occipital, and Mid-cerebellar Oscillations

To determine whether signals were specific to electrode locations Cz and Cbz, we assessed differences across the groups for the electrodes surrounding mid-frontal Cz (left motor cortical C3 and right motor cortical C4) and the electrode above Cbz (mid-occipital Oz). Strikingly, no main effects of “group” in the theta-band were observed over C3 [*F*_(2,33)_ = 2.41, *p* = 0.11, η^2^ = 0.13] or C4 [*F*_(2,33)_ = 1.9, *p* = 0.16, η^2^ = 0.1], or Oz [*F*_(2,33)_ = 2.74, *p* = 0.08, η^2^ = 0.14]. Additionally, no effects were observed in the beta-band for electrodes C3 [*F*_(2,33)_ = 0.16, *p* = 0.85, η^2^ = 0.01], C4 [*F*_(2,33)_ = 0.47, *p* = 0.63, η^2^ = 0.03], or Oz [*F*_(2,33)_ = 1.12, *p* = 0.34, η^2^ = 0.06]. Spectral power topographic plots for each group and frequency band can be observed in Supplementary Figure 3. These results clearly demonstrate the specificity of group differences to the mid-frontal and mid-cerebellar locations and demonstrate that the effects were independent from the surrounding electrodes.

To further distinguish mid-cerebellar activation from the surrounding muscles within the theta-band, we assessed differences between the Cbz electrode and surrounding EMG leads by assessing correlation coefficients (mean ± standard deviation = −0.007 ± 0.02) and zero-lag cross correlations (mean ± standard deviation = 0.00004 ± 0.001; Supplementary Figure 4). Similarly, cross-spectrum phase analyses (mean ± standard deviation = −2.3 ± 7) and cross-correlation analyses (mean ± standard deviation = −5,082.6 ± 70,670.8) were performed to assess similarities between Cbz and the nearby Oz electrode (Supplementary Figure 5). Even though in close proximity to the neck muscles and the Oz electrode, Cbz theta-band activity exhibited distinct activation patterns, as evidenced by the low cross-correlation between electrode Cbz and nearby EMG signals. Additionally, we performed a rmANOVA using electrodes Cbz, Oz, and Cz as within-subjects variables and groups PDPI+, PDPI–, and controls as between-subjects variables to assess theta-band power. The main effect was attributed to “electrode” [*F*_(2,33)_ = 32.27, *p* < 0.001, η^2^ = 0.34] and “group” [*F*_(2,33)_ = 8.48, *p* = 0.001, η^2^ = 0.19], but there was no interaction between the variables [*F*_(2,33)_ = 1.27, *p* = 0.292; Supplementary Figure 6]. Overall, our results describe distinct mid-frontal and mid-cerebellar theta-band changes in PDPI+ participants.

### Postural Control vs. Resting State

A remaining question was whether these mid-frontal and mid-cerebellar changes were specifically related to the postural task or whether they persisted during rest (Supplementary Figure 7). In contrast to aforementioned correlations in the postural task, a one-way ANOVA examining our mid-frontal site in the resting condition showed no main effect of “group” for the theta-band [*F*_(2,33)_ = 0.84, *p* = 0.44, η^2^ = 0.05] or for the beta-band [*F*_(2,33)_ = 0.96, *p* = 0.39, η^2^ = 0.06]. Similar to the mid-frontal site, no main effects of “group” were observed at the mid-cerebellar site at rest for theta [*F*_(2,33)_ = 1.27, *p* = 0.29, η^2^ = 0.07] and beta [*F*_(2,33)_ = 0.97, *p* = 0.39, η^2^ = 0.06].

Additionally, we performed rmANOVAs with “task” (rest vs. balance task) as within-subjects variables and group as between-subjects variables to assess theta-band and beta-band power. In the mid-frontal theta-band, we found a main effect of “task” (F_(1,33)_ = 12.34, *p* = 0.001, η^2^ = 0.23], no main effect of “group” [*F*_(2,33)_ = 1.67, *p* = 0.20, η^2^ = 0.05], and a significant interaction [*F*_(2,33)_ = 7.10, *p* = 0.003]. In contrast, the mid-frontal beta-band showed no main effect of “task” [*F*_(1,33)_ = 1.83, *p* = 0.19, η^2^ = 0.04], no main effect of “group” [*F*_(2,33)_ = 1.29, *p* = 0.29, η^2^ = 0.06], and no interaction [*F*_(2,33)_ = 0.04, *p* = 0.96]. In the mid-cerebellar theta-band, we found no main effect of “task” [*F*_(1,33)_ = 0.01, *p* = 0.94, η^2^ = 0.00] or “group” [*F*_(2,33)_ = 0.52, *p* = 0.6, η^2^ = 0.01], but we did observe an interaction [*F*_(2,33)_ = 5.23, *p* = 0.01]. In the mid-cerebellar beta-band, a trending main effect of “task” was observed [*F*_(1,33)_ = 3.95, *p* = 0.055, η^2^ = 0.07], but no main effect of “group” [*F*_(2,33)_ = 1.25, *p* = 0.3, η^2^ = 0.06] or interaction [*F*_(2,33)_ = 0.56, *p* = 0.57]. Overall, these analyses demonstrate that the specific theta band activity was proprietary to the postural control task and could not be observed in a similar manner during the resting state.

## Discussion

In this study, we demonstrated specific mid-frontal and mid-cerebellar oscillatory responses to postural control in PDPI+, PDPI−, and age-matched healthy control participants. There was a prominent decrease of theta oscillation power in the mid-frontal Cz and mid-cerebellar Cbz regions during the postural control task in PDPI+ participants, indicating a negative correlation between theta oscillations and postural control behavioral outcomes among PD patients. Our behavioral measures and mid-frontal/mid-cerebellar oscillations did not show differences between PDPI− and healthy control participants, pointing toward normal cortical and cerebellar neural networks regulating postural control in those patients.

In addition, we examined the effect of cognitive function in postural control *via* the DCCS task and found that PDPI+ participants performed the task poorly, compared to PDPI− and controls. Similar to our results, previous reports have shown a reduction in cognitive function in PD patients with abnormal balance and postural control (48, 49), suggesting a dysfunction of common neural networks in PDPI+. Furthermore, this relationship is supported by previous studies' results with lower theta activity in the frontal region at the occurrence of target stimuli during a cognitive task (24, 26, 50). Given the task-switching nature of the DCCS task, performance decreases seen in PDPI+ may reflect similar cognitive deficits during dual task interference, which has been shown in PD patients with postural instability during the performance of dual cognitive and motor tasks (51). Overall, both our and previous research suggest that cognitive function can be one of the non-motor features influencing postural control in PD. Moreover, a relationship between cognitive impairments and risk factors for falls have been demonstrated in PD patients (7, 49), as well as between postural instability and risk factors for falls (12, 52). Altogether, our and other authors' findings suggest that postural control in the presence of cognitive challenges may be one of the major contributors to PD falling.

Recently, body-worn triaxial accelerometers have been used by researchers to investigate postural control in human participants. The measurements computed from the accelerometer signals have been shown to be reliable and consistent with measurements computed from center of pressure data using traditional force platforms (53). Accelerometer outcomes are superior compared to traditional clinical assessments, because signals are collected continuously during the experiment, allowing researchers to examine time-dependent changes in postural control. In this study, we captured acceleration signals to demonstrate the time-dependent changes in postural control in all three groups and found poor postural measurements in PDPI+ participants. A prior report has shown that evaluation of time-dependent changes provides useful insights into postural control in PD and the effectiveness of intervention (44). Similarly, previous reports have shown poor postural control measurements in PDPI+ *via* different postural and balance measurement methods (12, 54).

In addition to accelerometer measures, this study showed that decreased mid-frontal and mid-cerebellar theta oscillations are related to postural instability in PD patients and demonstrated that increased mid-frontal and mid-cerebellar theta oscillations might be a possible defining feature of sufficient postural control. Our study adds further support to the increasing number of reports emphasizing the involvement of theta oscillations in postural control (55, 56). Similarly, our previous report demonstrated that lower mid-frontal theta activity was related to poor lower-limb motor performance in PD patients with freezing of gait (FoG) (25). In conjunction with the present results, the presence of postural instability in PD patients might be related to FoG (57) and possibly decreased theta activity in the respective areas during FoG events. Our results are consistent with a prior report about increased mid-frontal theta power as a necessary feature for optimizing postural control in healthy subjects (56).

Another previous study demonstrated increased theta activation in both sensorimotor and occipital areas of elderly people during a dynamic balance task. Theta activity further increased during a visual oddball cognitive task (58). In the same study, elderly people showed higher delta activity in the frontal region during a postural task. Altogether, it may be speculated that increased low-frequency oscillations in the frontal region may be network frequency bands specific to the processing of postural control in real-world balance conditions and during a dual balance-cognitive task. Noticeably, a prior report has shown that changes in cortical oscillations correlate with changes in surface stability in healthy young subjects and proposed that a relationship exists between mid-frontal theta oscillations and surface stability variation (59).

However, changes in theta oscillations were not restricted to the mid-frontal region but were also modulated in the mid-cerebellar region during the postural task in PDPI+ participants. This suggests an important role of theta oscillations in cortical and cerebellar regions to communicate information between the cortico-vestibular network and the cerebellum regarding postural control adjustments particularly through vestibulo-spinal and reticulo-spinal tracts (60). No theta modulations were observed in the lateral motor cortical electrodes (C3 and C4) near the hand areas, underlining that postural control might be associated with the tuning of a mid-frontal precentral leg area and mid-cerebellar theta network in the same frequency band through cortico-ponto-cerebellar circuits. Additional source localization techniques (e.g., MEG, BESA, and LORETA) could further differentiate this association, as mid-frontal Cz activity not only represents precentral First Motoneurons of the leg region for voluntary motion in a stationary task, but also other, possibly confounding premotor areas. Postural control in stance is modulated through multiple motor, premotor and prefrontal cortical areas, reticulospinal, and vestibulospinal tracts with mutual interconnectivity. Therefore, a direct functional association of the presented cerebellar theta activity with precentral motor activity should be taken with caution.

In this study, we also analyzed beta-band oscillations in the mid-frontal, motor cortical, and mid-cerebellar regions and found no changes in the power values between the groups, suggesting no significant contribution of the cortical and/or cerebellar beta network in the postural control task. However, the relationship between cortical and sub-cortical beta power and motor task performance has been well-studied in PD (47). Higher power in the beta-band was associated with poor lower-limb movement in PD patients with FoG (25, 61). These studies were consistent with the observation that functionally-related muscles share a common intermuscular beta-band input (62) and might be absent when there is no active movement. In the current study, PD participants were recorded with levodopa and our data showed no changes in beta power between all three groups during the resting condition, likely because dopaminergic therapy in PD normalizes the baseline cortical beta power in the resting-state (63).

Modulation of neural regions underlying postural control can be a potential alternative to normalize abnormal cortical and cerebellar oscillations and improve postural instability in PD patients. However, it remains unclear how non-invasive neuromodulation methods such as transcranial magnetic or electrical stimulation (TMS/tES) can effectively influence postural control in PD patients. Previous studies have shown that tDCS on cortical and/or cerebellar regions can influence balance control in PD patients (64, 65). TMS methods have also shown the potential to entrain oscillations in the target area and improve posture and balance control (66, 67). While modulations in cortical and cerebellar oscillations, specifically in the theta-band, are suggested to reflect adjustments in cortical and associated sub-cortical and cerebellar resources invested during postural control, the entrainment in theta oscillations *via* rTMS or tACS methods may indicate an improvement in overall cortico-cerebellar information transmission during postural control in PDPI+. Another pharmacotherapeutic approach to cerebellar activity modulation has been established in vertigo and nystagmus disorders, where 4-Aminopyridine proved to be a valuable attenuator of vertical nystagmus disorders (38). On the basis of the current results, further studies will have to investigate how possibilities of direct or pharmacological neuromodulation may alleviate postural instability in PD.

We should also acknowledge methodological limitations to this study. EEG signals contain low spatial resolution, so the detailed location of the mid-cerebellar activity within the brain could not be confirmed. However, ICA was applied to minimize volume conduction effects and we also demonstrated that the mid-cerebellar activity was different from mid-occipital activity during the postural control task. The signal comparisons in the theta-band between mid-cerebellar and mid-occipital regions showed dispersed correlation and phase, suggesting a difference in both signals. Moreover, we compared the mid-cerebellar EEG and nearby muscle EMG signals in the theta-band and observed discrete activation, suggesting no contamination of muscles to the mid-cerebellar EEG signals. However, future research would benefit from simultaneously using magnetoencephalography (MEG) and EMG recordings during postural control tasks and using source localization to confirm the exact location of these mid-cerebellar findings and their mid-frontal counterparts. However, the complexity of the shielded MEG setup is severely limited when measuring activity in the postural context, with wearable and moving systems (68) only in early stages of development. Their application in the conditions presented here may provide future additional insight. In addition, distributed and discrete EEG source localization like LORETA or BESA use mathematical approximations to overcome the spatial limitations of scalp EEGs at the cost of possible signal distortion. Given their complexity, these techniques are beyond the purview of this current pilot study.

In this study, we did not examine balance symmetry, since it can contribute to severe postural instability or balance control and FoG in PD patients (57). Moreover, previous studies have shown the role of cortical and associative networks in postural corrections or anticipatory/compensatory postural control (69, 70). As such, we cannot rule out the involvement of postural correction mechanisms or muscular activity related to anticipatory and compensatory reflexes needed to maintain balance among the recorded cortical and cerebellar oscillations. Furthermore, since PD patients in this study were subdivided into relatively small groups, future research would benefit from utilizing larger sample sizes to further determine the generalizability of these findings to larger subsets of the PD patient population and determine the exact contribution of important factors such as levodopa dose and disease progression. The setup of this study used a momentary analysis of subjects along their individual disease progression. One limitation of this approach is that PDPI− subjects may exhibit PDPI+ features later in their individual disease course. Therefore, a causative association of EEG features with variable disease progression cannot be established given the purview of the study setup. A follow-up study investigating current PDPI− subjects years later in their disease progression when they exhibit PDPI+ features would be beneficial to further support the current hypothesis. Finally, though we examined differences across specific frequency bands, future research may also benefit from examining slope differences across the entire power spectrum.

## Data Availability Statement

The raw data supporting the conclusions of this article will be made available by the authors, without undue reservation.

## Ethics Statement

The studies involving human participants were reviewed and approved by the University of South Dakota and the University of Iowa. The patients/participants provided their written informed consent to participate in this study.

## Author Contributions

TB, SK, CG, ML, EH, PB-P, and AS conceived the project. TB, SK, CG, and AS performed the analyses. TB, SK, CG, ML, EH, PB-P, and AS wrote and reviewed the manuscript. All authors contributed to the article and approved the submitted version.

## Funding

TB and AS were supported by the Division of Basic Biomedical Sciences and Center for Brain and Behavior Research (CBBRe) at the University of South Dakota, Vermillion, SD, USA.

## Conflict of Interest

The authors declare that the research was conducted in the absence of any commercial or financial relationships that could be construed as a potential conflict of interest.

## Publisher's Note

All claims expressed in this article are solely those of the authors and do not necessarily represent those of their affiliated organizations, or those of the publisher, the editors and the reviewers. Any product that may be evaluated in this article, or claim that may be made by its manufacturer, is not guaranteed or endorsed by the publisher.
