# Supplementary material for: Cortical and Cerebellar Oscillatory Responses to Postural Instability in Parkinson's Disease
Source: Front Neurol. 2021 Nov 4;12:752271. doi: 10.3389/fneur.2021.752271 (PMC8599431; doi:10.3389/fneur.2021.752271)
Supplement: Supplementary file 1 [file Data_Sheet_1.pdf]

Supplementary Information

Cortical and cerebellar oscillatory responses to postural instability in Parkinson's disease

Taylor J. Bosch,[1,2] Stefan Kammermeier,[3] Christopher Groth,[4] Matt Leedom,[5] Elizabeth K. Hanson,[6] Patti Berg-Poppe,[7] Arun Singh,[1,2,*]

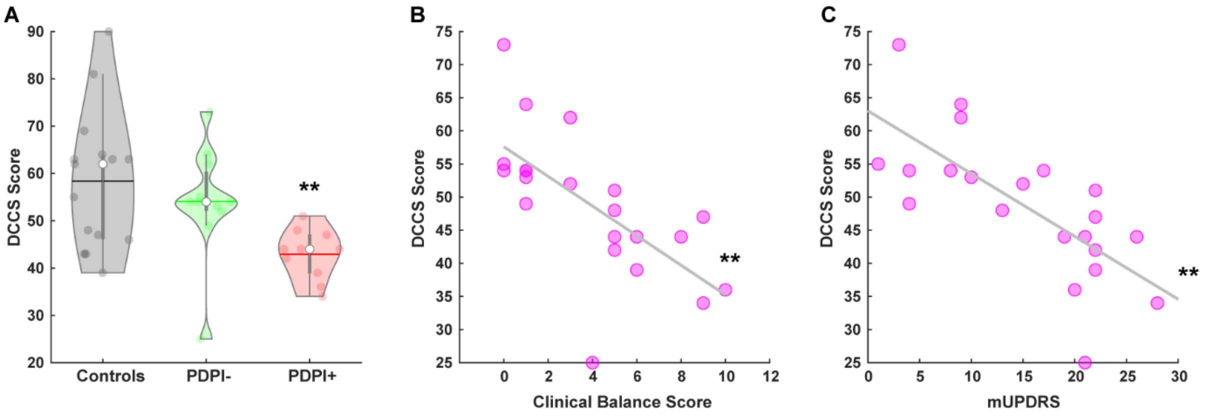

Supplementary Figure 1. Parkinson's disease patients with postural instability (PDPI+) show decreased cognitive function. (A) Performance on the Dimensional Change Card Sort (DCCS) test is significantly worse in PDPI+ compared to PDPI– and healthy controls. Worse DCCS performance (lower score) is associated with (B) worse clinical balance scores (higher score) and (C) increased disease severity as measured by the motor portion of the Unified Parkinson's Disease Rating Scale (mUPDRS). A) **$p = 0.01$ vs. controls. B-C) **$p < 0.01$. The horizontal lines and white circles in the violin plots represent the mean and median values, respectively.

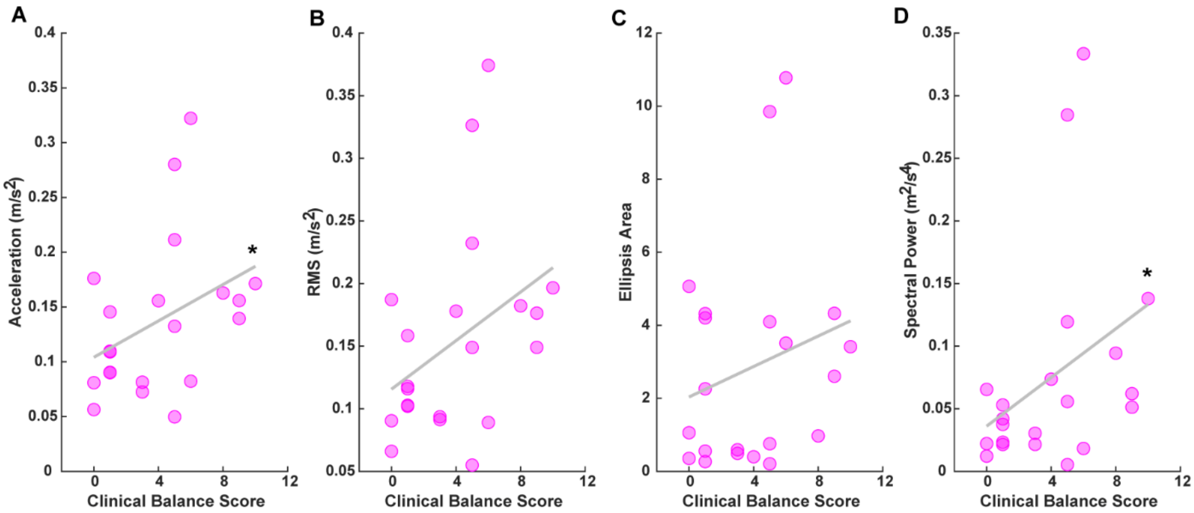

Supplementary Figure 2. Clinical balance scores are associated with postural stability accelerometer measures. (A) Higher clinical balance scores (indicating worse clinical balance) are associated with higher mean acceleration and (B) higher magnitude of acceleration. (C) Clinical balance scores are not associated with the area of the ellipsis computed using mean anteroposterior and mediolateral movement distances. (D) Higher clinical balance scores are associated with higher spectral power of acceleration across the 0.1-3 Hz frequency band. *$p < 0.05$, uncorrected.

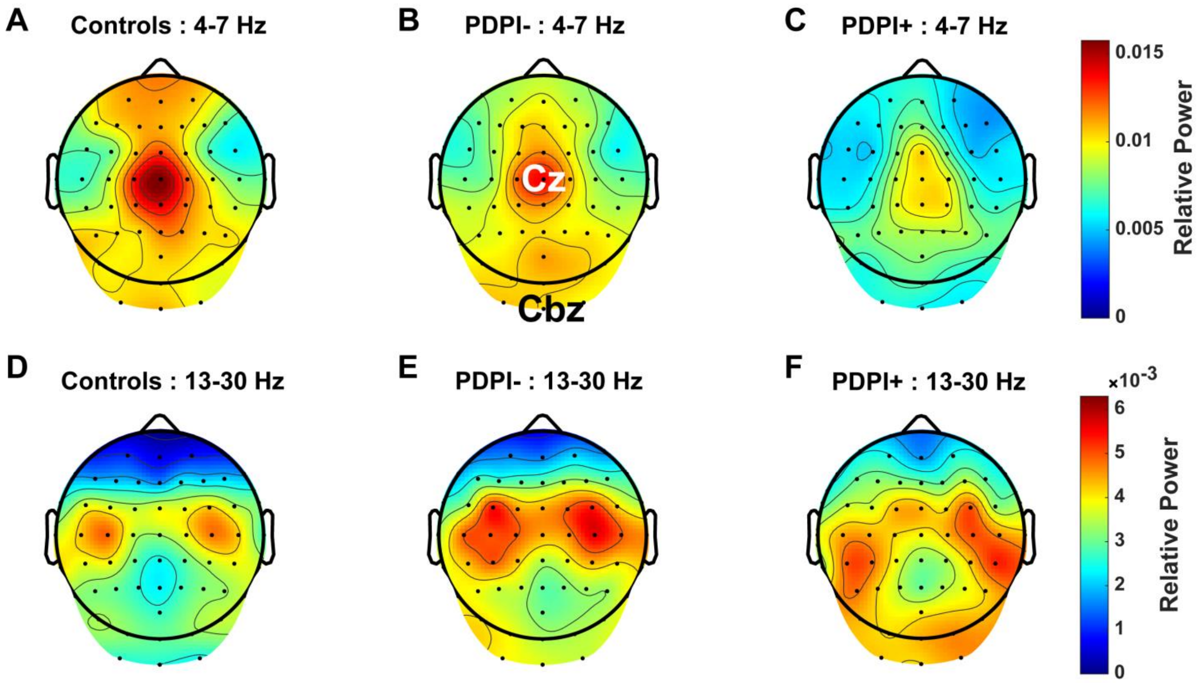

Supplementary Figure 3. Topographical maps demonstrating EEG signal scalp distributions. (A-C) Scalp distribution of relative power in the theta-band (4-7 Hz) for healthy controls, PDPI–, and PDPI+. (D-F) Scalp distribution of relative power in the beta-band (13-30 Hz) for healthy controls, PDPI–, and PDPI+. Locations of mid-frontal (Cz) and mid-cerebellar (Cbz) electrodes are labeled in panel B.

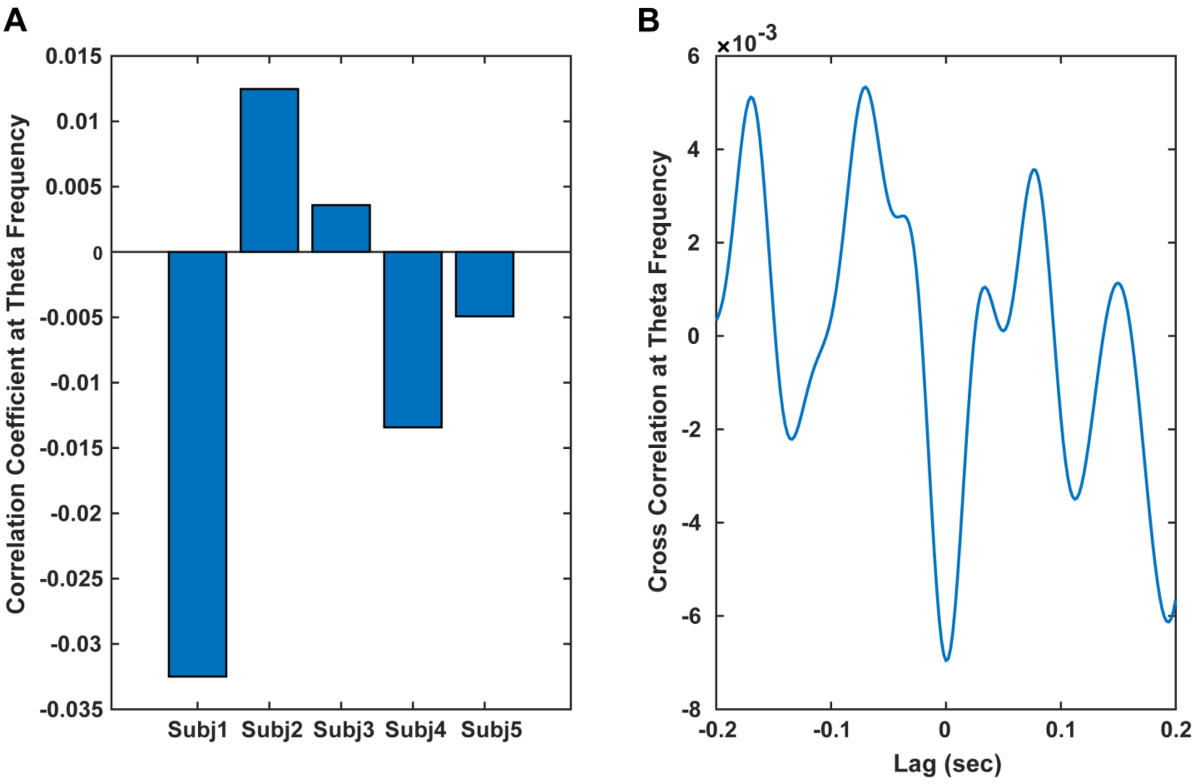

Supplementary Figure 4. Distinctions between mid-cerebellar theta-band activation and nearby muscle activity. (A) Correlation coefficients between the mid-cerebellar Cbz EEG electrode and surrounding EMG electrodes in the theta-band (4-7 Hz). (B) Cross correlation analyses examining zero-lag relationships between the mid-cerebellar Cbz electrode and surrounding EMG electrodes in the theta-band.

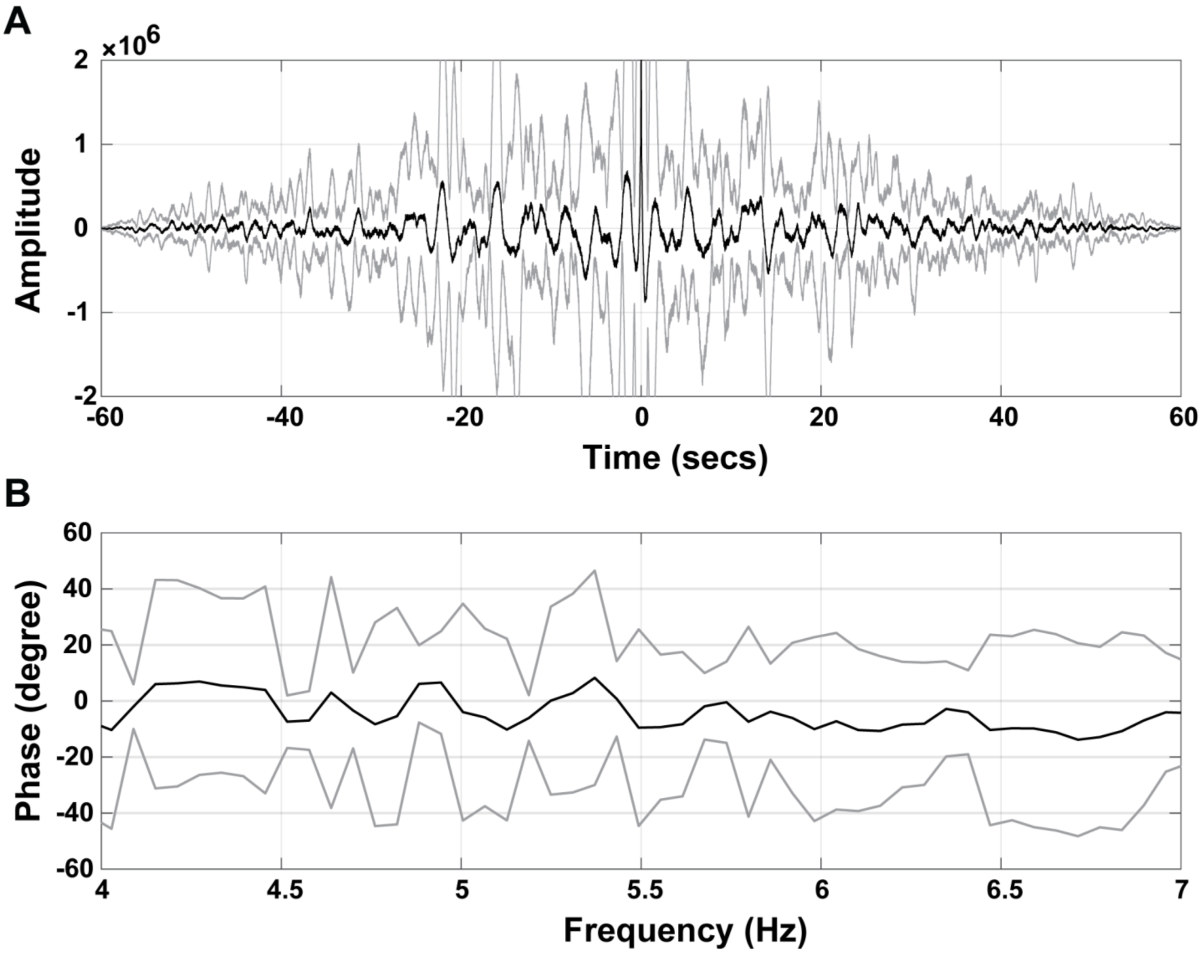

Supplementary Figure 5. Distinctions between mid-cerebellar and mid-occipital theta-band activation. Cross-correlation analyses were used to contrast the signal similarities between mid-cerebellar and mid-occipital theta-band signals using (A) amplitude and (B) phase values. Grey bars represent variance.

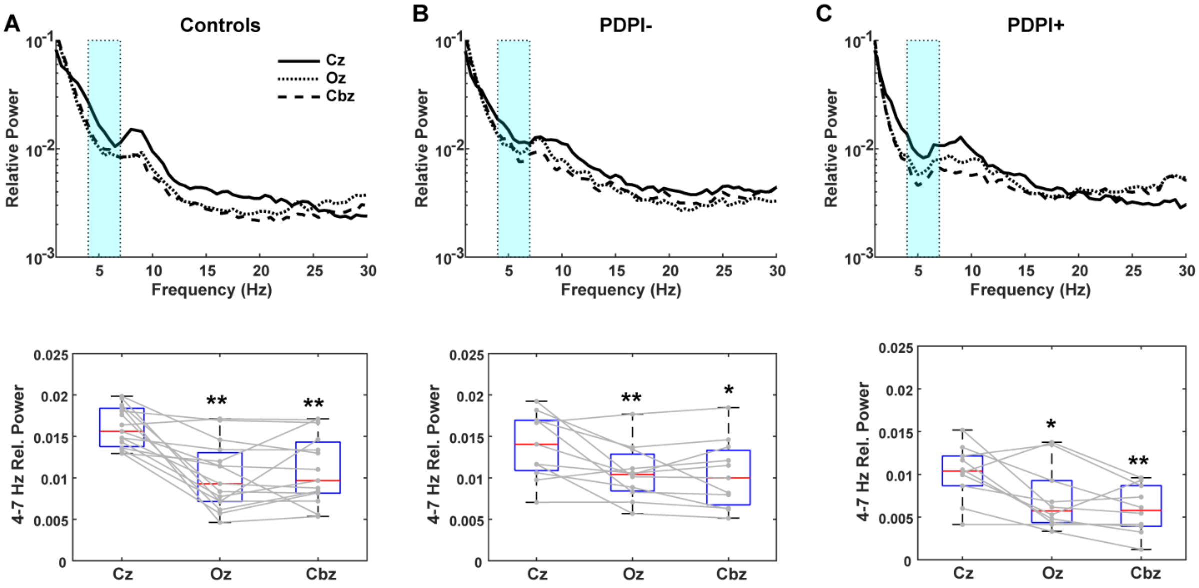

Supplementary Figure 6. Differences between mid-frontal, mid-occipital, and mid-cerebellar theta-band power across groups. Mid-frontal theta-band power was significantly different between mid-occipital and mid-cerebellar power in (A) healthy controls, (B) Parkinson's disease patients without postural instability (PDPI–), and (C) Parkinson's disease patients with postural instability (PDPI+). A-C: Cyan box indicates the theta-band. *p < 0.05 vs. Cz, **p < 0.01 vs. Cz.

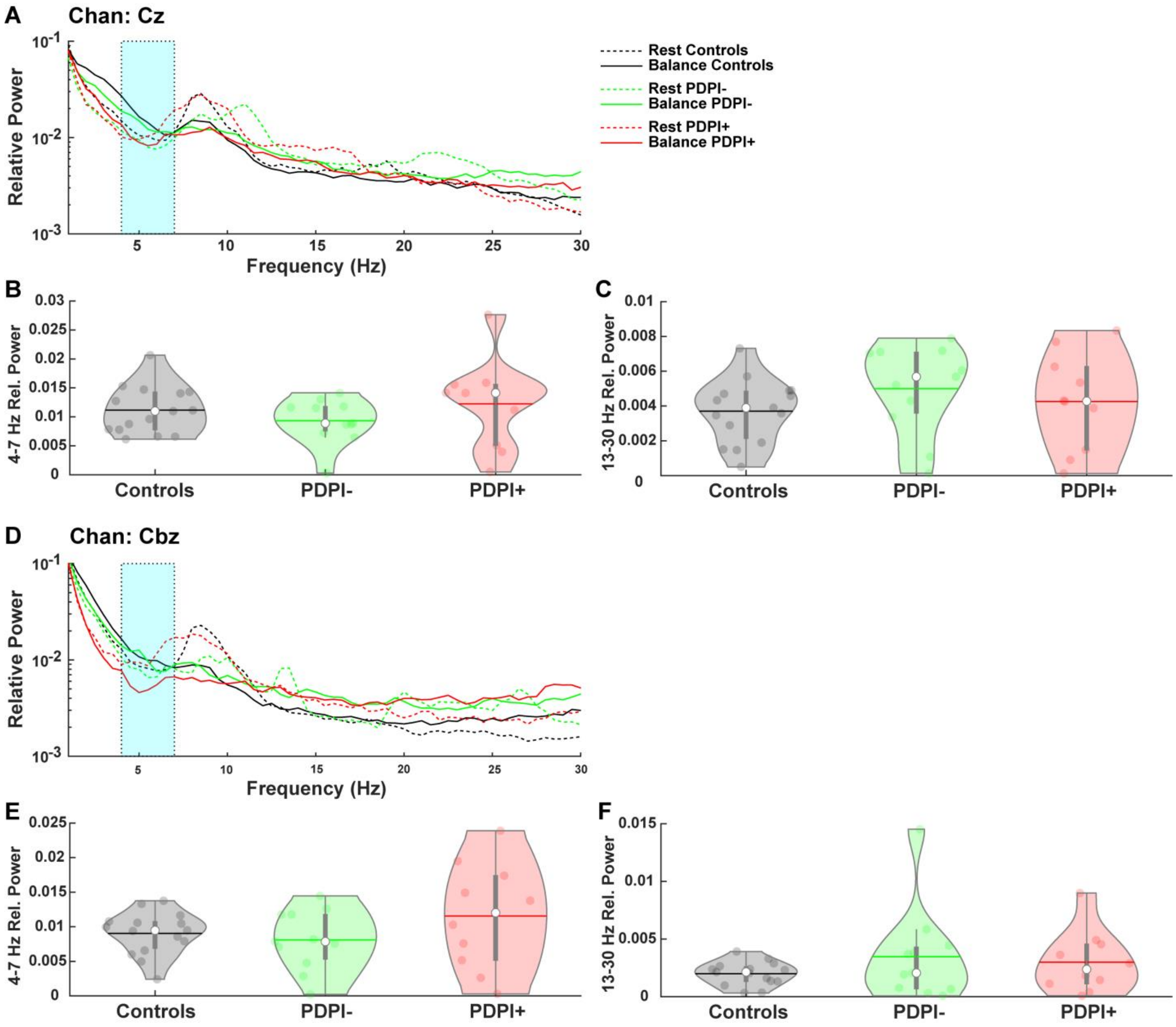

Supplementary Figure 7. Changes during the postural control task in mid-frontal and mid-cerebellar theta-band activity do not occur during resting-state. (A) Spectral power distribution at the mid-frontal Cz electrode for each group (controls, PDPI–, and PDPI+) during the postural control task and resting-state. No mid-frontal differences were observed across groups during the resting-state in (B) the theta-band (4-7 Hz) or (C) the beta-band (13-30 Hz). (D) Spectral power distribution at the mid-cerebellar Cbz electrode for each group (controls, PDPI, and PDPI+) during the postural control task and resting-state. No mid-cerebellar differences were observed across groups during the resting-state in (E) the theta-band or (F) the beta-band. A and D: Cyan box indicates the theta-band. The horizontal lines and white circles in the violin plots represent the mean and median values, respectively.
